# Successful Intravenous Thrombolysis in Ischemic Stroke Caused by Tuberculous Meningitis: A Case Report

**DOI:** 10.3389/fneur.2021.676684

**Published:** 2021-09-24

**Authors:** Xinke Peng, Xiaomei Wu, Lingling Lv, Qile Xiao, Yajing Zhan, Chunyu Wang, Hainan Zhang

**Affiliations:** Department of Neurology, The Second XiangYa Hospital of Central South University, Changsha, China

**Keywords:** stroke, tuberculous meningitis, thrombolysis (tPA), neurological deficits, vasculitis

## Abstract

Tuberculous meningitis (TBM) has a variety of clinical manifestations and complications, and ischemic stroke is a common complication of TBM. However, there is no established prevention or treatment for stroke associated with TBM, and the safety and efficiency of thrombolysis in acute stroke caused by TBM remain unknown. Herein, we present a case of successful intravenous thrombolysis in ischemic stroke caused by TBM. A 50-year-old male patient with cerebral infarction had substantially improved neurological function after intravenous thrombolysis, and he was subsequently found to have TBM. Our findings suggest that intravenous thrombolysis might be an effective acute treatment method for infectious stroke.

## Introduction

Tuberculous meningitis (TBM), an important cause of infectious stroke, is the most devastating form of extrapulmonary tuberculosis (1, 2). Intravenous thrombolysis is the recommended therapy for acute ischemic stroke in adults within 4.5 h of the onset of symptoms. However, there are few clinical reports regarding the safety and outcomes of thrombolysis treatment for TBM. This case report describes a patient with cerebral infarction who had substantially improved neurological function after intravenous thrombolysis. The patient was subsequently diagnosed with TBM during the evaluation process to determine the mechanism of his stroke.

## Case Report

A 50-year-old man was admitted to our emergency department with sudden-onset right-sided weakness and aphasia that occurred 3 h ago. In the past month, the patient had headaches, manifested by paroxysmal pain in both temples and the base of the neck. He had a history of fever and chills and vomited 3 days ago. His initial National Institutes of Health Stroke Scale (NIHSS) score was 10 based on analyses of drowsiness, global aphasia, dysarthria, right-sided hypoesthesia, and facial nerve paralysis.

**Figure 1:**
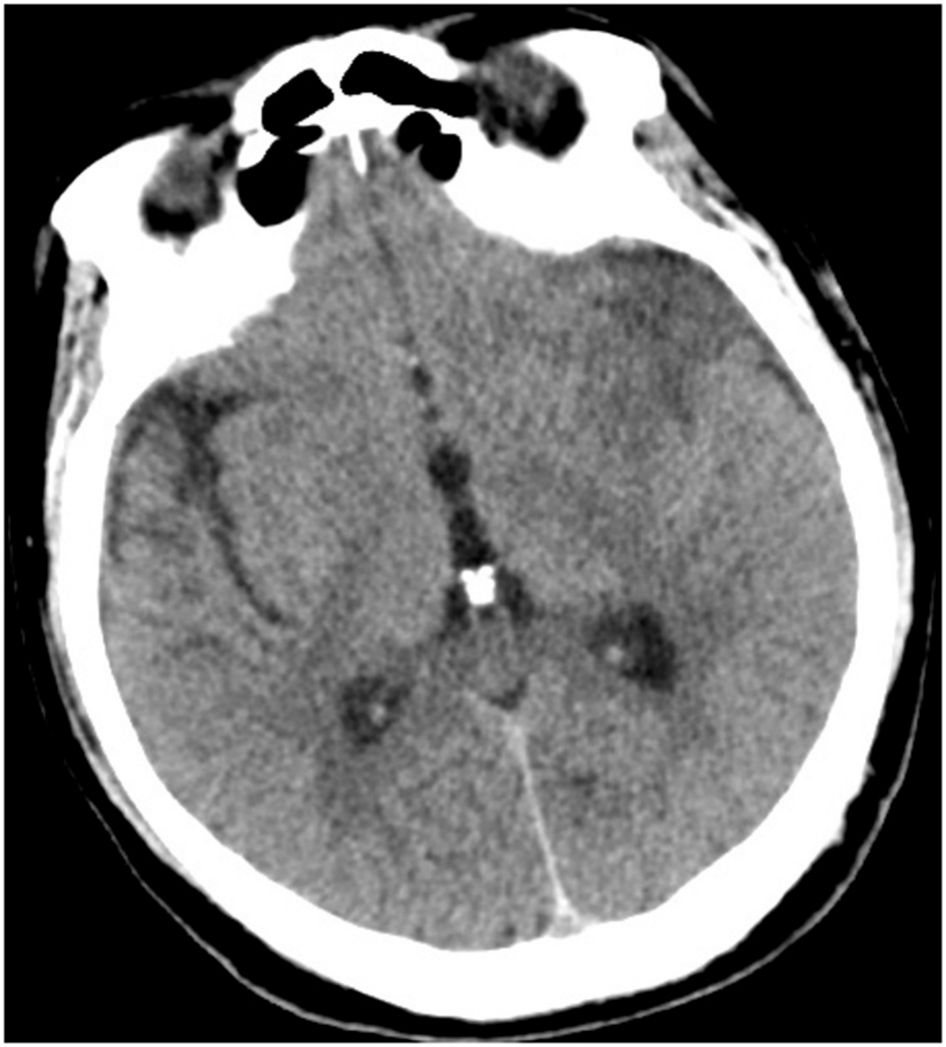
Initial head CT revealed left frontal lobe and left insular lobe hypodensities.

**Figure 2:**
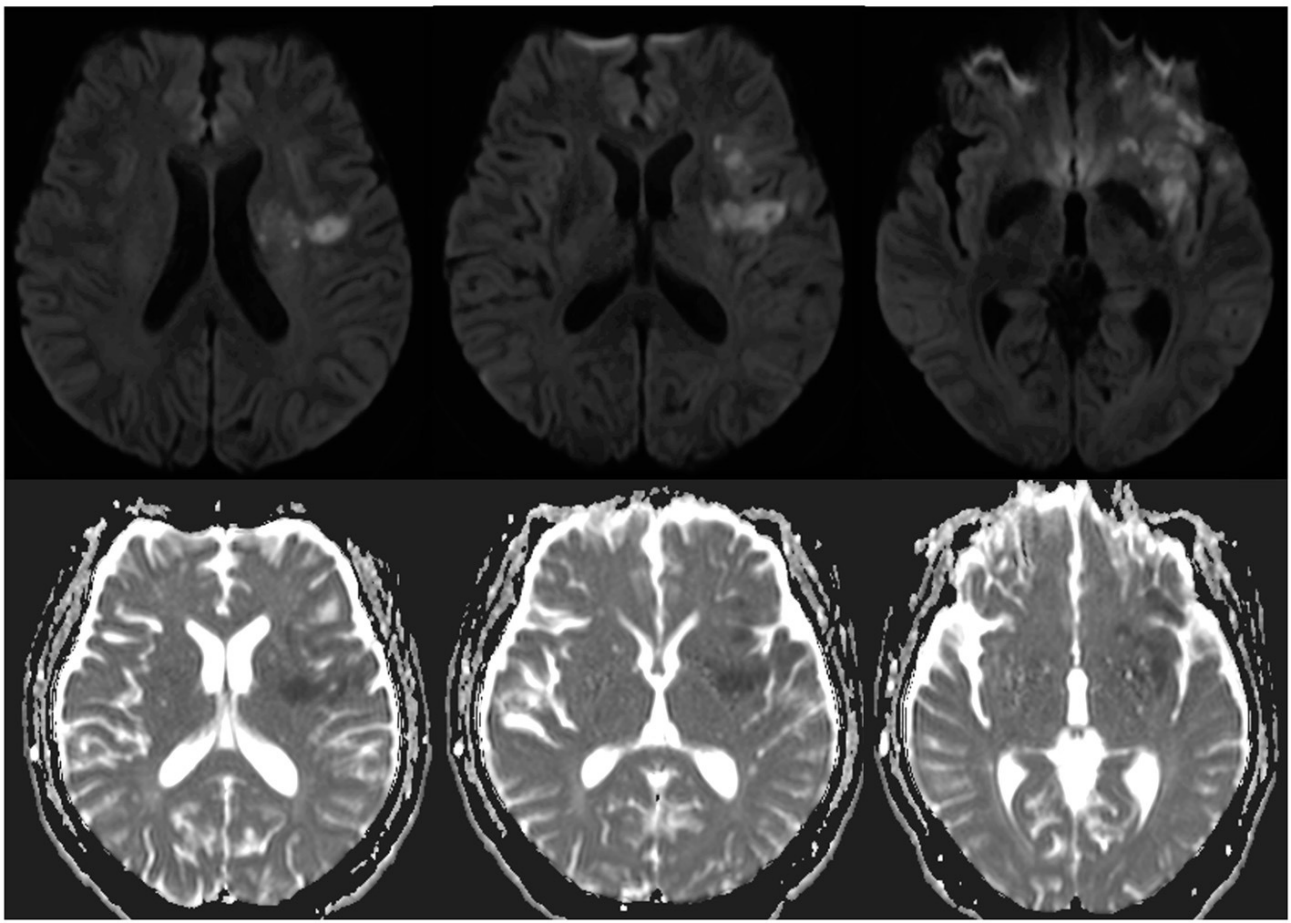
Axial diffusion-weighted image and apparent diffusion coefficient showed acute infarction in the left temporal lobe, left insular lobe, left basal ganglia, and left radial crown 24 h after admission.

**Figure 3:**
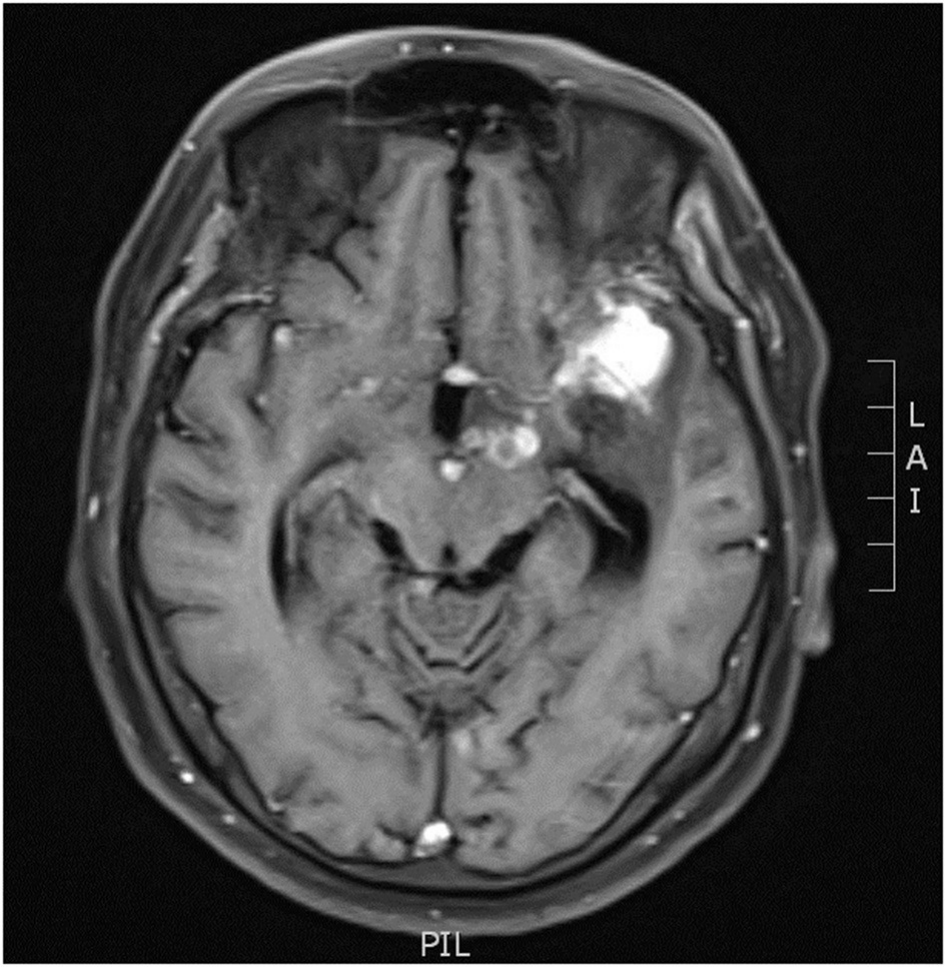
MRI revealed nodular enhancement in the left pontine cistern.

Initial laboratory examinations showed a white blood cell (WBC) count of 12.56 × 109/L (neutrophils, 88.2%), blood sodium level of 124.2 mmol/L, and chlorine level of 88.2 mmol/L. Initial non-enhanced head computed tomography (CT) revealed no hemorrhage but showed hypodensity throughout the left frontal lobe and left insular lobe, indicating acute ischemia (Figure 1). Transthoracic echocardiography showed no obvious neoplasms, and cerebral infarction caused by infective endocarditis was thus not considered. After excluding absolute contraindications for intravenous thrombolysis, the patient was treated with an intravenous recombinant tissue plasminogen activator (rt-PA). His neurological defects improved substantially after thrombolytic therapy. Twenty-four hours after rt-PA administration, he had only mild facial paralysis on the right side, and his reevaluated NIHSS score was only 1. Post-rt-PA magnetic resonance imaging (MRI) found multiple cerebral infarctions (Figure 2), and contrast-enhanced MRI revealed that the hypodense area in the left insula and frontal lobe was edema adjacent to a tuberculoma but not ischemia (Figure 3). The patient did not complete brain CT angiography (CTA) and CT perfusion (CTP). The initial hypodensity on the non-contrast CT scan, which had been misinterpreted as ischemic changes, did not match the patient's clinical symptoms. Chest CT showed a slight exudation of the double lower lungs and a few fibrous foci in the upper right and lower left lungs. The patient received empirical administration of piperacillin and tazobactam; however, his symptoms did not improve, and he continued to be intermittently febrile.

On the third day of admission, the patient developed lethargy. Considering his persistent fever and refractory hyponatremia since admission, the patient underwent a lumbar puncture with pressure > 40 cmH20. The cerebrospinal fluid (CSF) analysis revealed a WBC count of 66 × 106/L, lymphocyte count of 70%, protein level of 925.1 mg/L, glucose level of 3.0 mmol/L, and chloride level of 103.5 mmol/L. Findings of CSF cryptococcal antigen and bacterial and fungal cultures, as well as results of the purified protein derivation test and sputum cultures, were all negative. High-throughput sequencing of the CSF detected *Mycobacterium tuberculosis* with high confidence. Re-examination of head MRI enhancement revealed that the left pontine cistern showed nodular enhancement (Figure 3), and multiple cerebral infarctions with complicated TBM were diagnosed. The patient was thus started on a five-drug treatment for tuberculosis comprising isoniazid, ethambutol, pyrazinamide, rifampicin, and levofloxacin. Intrathecal administration of dexamethasone (4 mg/time) and isoniazid (100 mg/time) were given three times per week. Further CSF examinations showed improved tendencies (Table 1). The patient had only right facial paralysis when discharged, and he continued anti-tuberculosis treatment at a local hospital.

**Table 1:** Results of serial cerebrospinal fluid analyses.

	**4th day**	**9th day**	**29th day**	**41st day**	**51st day**
Pressure (cmH20)	>40	22.5	16	21	18
WBC (×10^6^/L)	66	140	62	33	0
Lymphocyte count (%)	30	60	10	10	0
Protein (mg/L)	925.1	476.6	598.0	982.1	497.2
Glucose (mmol/L)	3.0	2.4	3.6	3.3	3.0
Chloride (mmol/L)	103.5	115.8	113.7	117.3	117.6

## Discussion

TBM is the most life-threatening extrapulmonary tuberculosis due to its high mortality rate, and stroke may occur in 15−57% of TBM cases (1). Cerebral infarction is a predictor of poor outcomes (3), and the mortality rate of TBM patients with stroke may be three times that of patients without infarction (4). The extension of the inflammatory exudate along the perforating blood vessels into the brain substance causes vascular damage, which may lead to spasm or thrombosis of the vessels, with resulting ischemia or infarction (5). Most cerebral infarctions associated with TBM are multiple, and up to 75% of them are located in the basal ganglia, anteromedial thalamus, anterior limb, and genu of the internal capsule (6–8). Perforating arteries are most involved in cerebral infarctions caused by tuberculous cerebral arteritis; therefore, basal ganglia and internal capsule infarctions are the most common (9). Consistently, the patient in this study had multiple-site infarctions, further verifying that TBM is prone to cooccur with cerebral infarction. However, this patient had no history of tuberculosis before the onset of symptoms, which is usually uncommon.

Aspirin is the most commonly used treatment for ischemic stroke secondary to TBM because it can prevent stroke and modulate the host immune response in TBM patients (7, 8). However, aspirin is only used as a secondary prevention drug and cannot improve the symptoms of neurological deficits in stroke patients with intravenous thrombolysis. To date, no clear clinical guidelines recommend thrombolytic therapy for acute cerebral infarction secondary to TBM (7). According to the European Stroke Organization (ESO) guidelines on intravenous thrombolysis for acute ischemic stroke, infectious strokes, including infective endocarditis, are generally considered a relative contraindication to thrombolytic therapy (10). However, recently, Sloane et al. (11) analyzed 26 cases of infective endocarditis with endovascular recanalization treatment, and they found that only one case did not improve after successful removal of the thrombus. At the time of thrombolytic infusion, we were unaware that our patient had TBM. However, we noticed that the patient continued to have fever and headache, and we thus ruled out infective endocarditis in a limited time. Fortunately, this patient showed a good therapeutic effect after intravenous thrombolysis. We believe that our patient might have benefited from thrombolysis.

One limitation should be mentioned in this study. This study only analyzed one clinical case; thus, it is difficult to draw a firm conclusion, and further studies with a larger sample size are needed to confirm our present findings. Our findings suggest that in patients with acute cerebral infarction accompanied by headache and fever, the possibility of an infectious stroke should be considered, and intravenous thrombolysis might be an effective acute treatment method for patients with infectious stroke.

## Data Availability Statement

The original contributions presented in the study are included in the article/supplementary material, further inquiries can be directed to the corresponding author/s.

## Ethics Statement

Written informed consent was obtained from the individual(s) for the publication of any potentially identifiable images or data included in this article.

## Author Contributions

XW and YZ are the guarantor of integrity of the entire study. LL and QX performed data acquisition. XP was responsible for manuscript preparation and editing. CW and HZ performed manuscript review. All authors read and approved the final manuscript.

## Conflict of Interest

The authors declare that the research was conducted in the absence of any commercial or financial relationships that could be construed as a potential conflict of interest.

## Publisher's Note

All claims expressed in this article are solely those of the authors and do not necessarily represent those of their affiliated organizations, or those of the publisher, the editors and the reviewers. Any product that may be evaluated in this article, or claim that may be made by its manufacturer, is not guaranteed or endorsed by the publisher.
